# Neuroprotective Role of Selenium Nanoparticles Against Behavioral, Neurobiochemical and Histological Alterations in Rats Subjected to Chronic Restraint Stress

**DOI:** 10.1007/s12035-024-04196-3

**Published:** 2024-05-04

**Authors:** Sarah A. Elfakharany, Samir S. Eskaros, Nesrine M. El Azhary, Doaa A. Abdelmonsif, Teshreen M. Zeitoun, Gamal A. G. Ammar, Youssef A. Hatem

**Affiliations:** 1 Department of Medical Physiology, Faculty of Medicine, University of Alexandria, Al-Mouassat Medical Campus, El Hadara, Alexandria, Egypt; 2 Department of Medical Biochemistry, Faculty of Medicine, University of Alexandria, Al- Moussat Medical Campus, El Hadara, Alexandria, Egypt; 3 Department of Histology and Cell Biology, Faculty of Medicine, University of Alexandria, Al-Moussat Medical Campus, El Hadara, Alexandria, Egypt; 4 Biotechnology Unit, Plant Production Department (PPD), Arid Lands Cultivation Research Institute (ALCRI), City of Scientific Research and Technological Applications (SRTA-City), New Borg El‑Arab City, Alexandria, Egypt

**Keywords:** Chronic restraint stress, Selenium nanoparticles, Serotonin, Oxidative stress, TNF-α, Caspase-3

## Abstract

Chronic stress induces changes in the prefrontal cortex and hippocampus. Selenium nanoparticles (SeNPs) showed promising results in several neurological animal models. The implementation of SeNPs in chronic restraint stress (CRS) remains to be elucidated. This study was done to determine the possible protective effects of selenium nanoparticles on behavioral changes and brain oxidative stress markers in a rat model of CRS. 50 rats were divided into three groups; control group (*n* = 10), untreated CRS group (*n* = 10) and CRS-SeNPs treated group (*n* = 30). Restraint stress was performed 6 h./day for 21 days. Rats of CRS-SeNPs treated group received 1, 2.5 or 5 mg/kg SeNPs (10 rats each) by oral gavage for 21 days. Rats were subjected to behavioral assessments and then sacrificed for biochemical and histological analysis of the prefrontal cortex and hippocampus. Prefrontal cortical and hippocampal serotonin levels, oxidative stress markers including malondialdehyde (MDA), reduced glutathione (GSH) and glutathione peroxidase (GPx), tumor necrosis factor alpha (TNF-α) and caspase-3 were assessed. Accordingly, different doses of SeNPs showed variable effectiveness in ameliorating disease parameters, with 2.5 mg/kg dose of SeNPs showing the best improving results in all studied parameters. The present study exhibited the neuroprotective role of SeNPs in rats subjected to CRS and proposed their antioxidant, anti-inflammatory and anti-apoptotic effects as the possible mechanism for increased prefrontal cortical and hippocampal serotonin level, ameliorated anxiety-like and depressive-like behaviors and improved prefrontal cortical and hippocampal histological architecture.

## Introduction

Recently, mankind is experiencing different sets of circumstances that lead to diverse types of stress. Stress is the adverse stimuli boosting a negative impact on the body homeostasis, with the resultant physiological and psychological responses [1, 2]. Physiological responses to stress occur via activation of sympatho-adrenomedullary (SAM) axis and hypothalamic pituitary adreno-cortical (HPA) axis. SAM axis comprises the rapid, immediate response to stress in which there is release of epinephrine and norepinephrine from the adrenal medulla and release of norepinephrine from the sympathetic nerves [3, 4]. HPA axis comprises the slower, prolonged response to stress in which there is release of corticotropin-releasing hormone (CRH) from the hypothalamus. CRH induces adrenocorticotropic hormone (ACTH) production from the anterior pituitary gland. Then, ACTH stimulates glucocorticoids secretion from the adrenal cortex [3].

Exposure to chronic stress results in overactivation of HPA axis. This leads to sustained elevation in the glucocorticoids level, which induces structural and functional changes in the brain, specifically the prefrontal cortex and hippocampus. These regions regulate behavior and emotions, and process psychogenic stress [5]. Oxidative stress is the imbalance between reactive oxygen species (ROS) production and antioxidant defense systems [6]. The brain is highly susceptible to oxidative stress, due to its high oxygen consumption, high lipid content and low antioxidant levels [7]. It was reported that neuronal oxidative stress develops as a consequence of chronic psychological stress, with its resultant DNA damage, lipid peroxidation and alterations in proteins functioning [8]. It was evidenced that chronic psychological stress results in activation of brain microglia that release large quantities of pro-inflammatory cytokines, such as tumor necrosis factor-alpha (TNF-α) [9]. Chronic stress is capable of upregulating the expression of pro-apoptotic proteins, such as caspase-3 [10]. In chronic psychological stress, release of neurotransmitters that regulate mood and behavior, such as serotonin is greatly diminished [11]. Animal models of chronic stress enabled better understanding of its effects on brain morphology and functioning. Chronic restraint stress (CRS) model is utilized in preclinical studies to demonstrate the effects of chronic psychological stress [12]. It leads to behavioral alterations that resemble day to day stressors that are repeated and piled up on the previous day’s workload [13].

Nowadays, nanomedicine field is gaining popularity for enabling the implementation of nanostructures in diagnosing, preventing and treating various diseases [14]. Nanomaterials have small sizes that vary between 1 and 100 nm, and high surface area to volume ratio. Therefore, nano-based drug delivery systems provide better absorption, bioavailability and stability than other known drug delivery systems [15]. Selenium nanoparticles are a subject of interest in nanomedicine for multiple reasons. First, they have higher uptake by the cells than selenium when taken orally as they are absorbed by endocytosis [14]. Second, they exhibit better antioxidant activity than any other chemical forms of selenium. They also have higher bioavailability than selenium due to the possibility of utilizing selenium in the zero-oxidation state (Se0) [16]. Third, they are far less toxic than selenium [17]. The possible neuroprotective effects of selenium nanoparticles in chronic stress remains to be elucidated.

## Material and Methods

### Experimental Animals

The study was carried out on 50 adult male Wistar rats aged 8–10 weeks, with a body weight ranging from 200–250 g. The animals were kept under standard laboratory conditions, maintained on a 12-h light–dark cycle with free access to food and water at the animal house of Physiology Department. All experimental procedures were conducted according to the NIH Guide for Care and Use of Laboratory Animals and followed the ARRIVE guidelines. The study protocol was approved by the institutional Medical Ethics Committee, Faculty of Medicine (IRB NO: 00012098; FWA NO: 00018699; approval NO: 0106414).

### Preparation of Selenium Nanoparticles (SeNPs)

Selenium nanoparticles were synthetized by polyethylene glycol (PEG)-200 according to Nie et al., 2016 with minor modifications. 170 mg selenium powder was added in 200 mL PEG-200 solution containing 0.1M glucose at 100°C for 20 min until the complete formation of the nanoparticles, under magnetic stirring. Glucose was used as a reducing agent, while polyethylene glycol acted as a surface decorator of selenium nanoparticles to enhance their stability [18].

To study shape and size of SeNPs, transmission electron microscopy (TEM; JEOL-JSM 1400 Plus, Tokyo, Japan) was done. Samples were prepared by placing a drop of the nanosuspension on paraffin sheet. Then, carbon coated grid was placed on the sample and left for 1 min to allow nanoparticles to adhere on the carbon substrate. The remaining suspension was removed and samples were air dried before microscopic examination [19]. Furthermore, particle size distribution, polydispersity index (PDI) and zeta potential of the dispersion were determined by dynamic light scattering (DLS) technique using Zetasizer Nano ZS. (Malvern, Instruments Ltd., Malvern, UK).

### Experimental Groups and Tissue Sampling

**Fig. 1:**
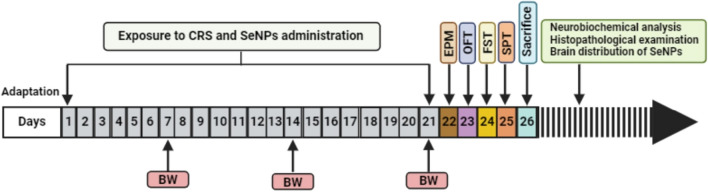
Schematic representation of the experimental design. CRS: chronic restraint stress, SeNPs: selenium nanoparticles, BW: body weight, EPM: elevated plus maze test, OFT: open field test, FST: forced swim test, SPT: sucrose preference test

After 7 days of adaptation, rats were divided into 3 groups as follows: *control group* (*n* = 10) in which rats received 1.5 ml distilled water daily by oral gavage for 21 days as a vehicle and were not exposed to chronic restraint stress; *untreated chronic restraint stress (CRS) group* (*n* = 10) in which rats were exposed to chronic restraint stress. Each rat was placed inside a small and narrow device in order to restrict its movement [20]. This device was in the form of a plastic bottle, whose dimensions were 21 (height) × 7 (diameter) cm. This was done daily over a period of 6 h. for 21 days from 9.00 am to 3.00 pm. These plastic bottles kept the rats well-contained with very limited movements and had holes to allow sufficient breathing [2]. During the restraint stress period, rats were deprived of food and water [21]. At the same time, each rat in this group also received 1.5 ml distilled water by oral gavage daily as a vehicle 15 min before the restraint stress period; *CRS- SeNPs treated group* (*n* = 30) which was subdivided into 3 subgroups (10 rats each) in which rats received different doses of SeNPs (1 mg/kg, 2.5 mg/kg or 5 mg/kg SeNPs) dissolved in 1.5 ml distilled water by oral gavage daily 15 min before the restraint stress period for 21 days [22]. Body weights of all rats were recorded at days 7, 14 and 21 of the study. Behavioral tests were conducted after day 21 of the study for 4 consecutive days. At day 26 of the study, rats were sacrificed by decapitation under ether anesthesia. The whole brain was removed immediately and rinsed in ice cold saline. The prefrontal cortical and hippocampal tissues were quickly dissected and stored at -80 °C until they were homogenized in the appropriate buffer for biochemical analysis. Prefrontal cortical and hippocampal tissue specimens from all studied groups were processed for histopathological examination. Another specimen of the prefrontal cortical and hippocampal tissues from the SeNPs-treated subgroups was assigned for transmission electron microscopic examination (TEM; JEOL-JSM 1400 Plus, Tokyo, Japan) to confirm the uptake of SeNPs (Fig. 1).

### Behavioral Tests

Behavioral tests were performed between 9.00 am and 5.00 pm, and were recorded and analyzed by an observer who was blind to the experimental groups.

1. Elevated plus maze test

This test was performed at day 22 of the study to evaluate anxiety-like behavior of rats. The maze consisted of two open arms perpendicular to two closed arms and a small central square between the arms. The maze was elevated 70 cm above the floor. Each rat was placed individually at the center of the maze with its head facing towards the open arm and was allowed to explore it freely for 5 min. Number of entries in closed arms, number of entries in open arms, time spent in closed arms and time spent in open arms were recorded. After each rat trial, the arena was cleaned with 70% ethyl alcohol [23].

2. Open field test

This test was performed at day 23 of the study to evaluate the locomotor activity and anxiety-like behavior of rats. The arena consisted of an open wooden box, whose dimensions were 75 (length) × 75 (width) × 35 (height) cm and its floor was divided into 16 squares. Each rat was placed individually at the center of the arena and was allowed to explore it freely for 5 min. Number of squares crossed and number of rears were recorded. After each rat trial, the arena was cleaned with 70% ethyl alcohol [24].

3. Forced swim test

This test was performed at day 24 of the study to evaluate behavioral despair. Rats were placed in an open plastic container, whose dimensions were 50 (height) × 40 (diameter) cm and was filled up to 40 cm of its height with water at 25 ± 1°C. Rats were trained to swim for 15 min. Twenty-four hours later, they were forced to swim again for 5 min. During this 5-min period, the immobility time was recorded. The rat was considered immobile if it was floating passively with only minor movements to keep its head above the water. After each trial, rats were dried with a towel and returned to their cages [25].

4. Sucrose preference test

This test was performed at day 25 of the study to evaluate anhedonia [26]. Anhedonia is defined as diminished capability to experience pleasure and it is a core symptom of depressive behavior [27]. This test required training. Each rat was placed alone in a small cage. In the first 24 h., each rat was presented with two bottles; both containing 100 ml of 2% sucrose solution. In the following 24 h., each rat was also presented with two bottles; one containing 100 ml of 2% sucrose solution and the other containing 100 ml of tap water. After training, rats were deprived of water for 16 h. Later on, the test was done where each rat was presented with a bottle containing 100 ml of 2% sucrose solution and another containing 100 ml of tap water for 4 h. After 2 h., the position of the bottles was changed to avoid side preference. After the 4 h. duration, the bottles were weighed and percentage of sucrose preference was calculated using the following formula: (sucrose solution consumption/sucrose solution consumption + water consumption) X 100 [28].

### Brain Distribution of SeNPs

Prefrontal cortical and hippocampal tissue specimens were first cut into (0.5–1 mm) cubes and were immediately fixed in 4% formaldehyde/1% glutaraldehyde. These cubes were post-fixed in 1% osmium tetroxide, rinsed in phosphate buffer for 1–2 h, dehydrated with graded ethanol, embedded in plastic capsules of araldite, polymerized, and processed to get semithin sections. The semithin sections were cut, stained with methylene blue and localized using a light microscope. Ultrathin sections were stained with uranyl acetate and lead citrate [29].

### Prefrontal Cortical and Hippocampal Tissues Neurobiochemical Assays

1. Determination of serotonin by high performance liquid chromatography (HPLC)

For that purpose, prefrontal cortical and hippocampal tissues were homogenized in ice cold methanol (4 ml/g tissue). Then, 1 ml of the homogenate was centrifuged at 14,000 rpm for 20 min. The supernatant was left to be evaporated to dryness by vacuum freeze. The dry residue was reconstituted with 300 µl de-ionized water and mixed with the vortex for 10 s. Afterwards, 300 µl solution of chloroform: isopropanol (100:30, v/v) was added and mixed with the vortex for 2 min. Then, it was centrifuged at 3000 rpm for 5 min. The upper aqueous layer was separated and injected into HPLC system [30]. For chromatographic separation, an HPLC Agilent 1100 chromatography system was used. Chromatographic separation was achieved on a Zorbax SB C18 chromatography column (4.6 × 250 mm, 5 µm). The mobile phase consisted of acetate buffer (pH 3.5, 12 mM acetic acid, 0.26 mM Na2EDTA)–methanol (86:14, v/v). The fluorescence was monitored at excitation and emission wavelengths of 279 nm and 320 nm, respectively. Peaks were recognized by comparing their retention time in the tissue sample extract solution with that of standard solution. The results were normalized to tissue weight to be expressed as ng/mg tissue [31].

2. Measurement of markers of oxidative stress state

Prefrontal cortical and hippocampal tissues were homogenized in 10 times (w/v) ice cold 0.1 M phosphate buffer (pH 7.4) containing protease inhibitor cocktail (Sigma-Aldrich, St. Louis, MO, USA) and centrifuged at 10,000 xg at 4°C for 15 min [19]. The quantitative measurement of the lipid peroxidation marker, malondialdehyde (MDA), reduced glutathione (GSH) and glutathione peroxidase (GPx) activity was performed by colorimetric assay according to the instructions provided by the kit (Sigma-Aldrich, St. Louis, MO, USA). MDA results were normalized to tissue weight to be expressed in nmol/g tissue while GSH results were normalized to tissue weight to be expressed in mg/g tissue. Finally, GPx activity results were normalized to tissue weight to be expressed in U/g tissue.

3. Measurement of tumor necrosis factor alpha (TNF-α) and caspase-3 by ELISA

Prefrontal cortical and hippocampal tissues were homogenized in 10 times (w/v) ice cold 0.1 M phosphate buffer (pH 7.4) containing protease inhibitor cocktail (Sigma-Aldrich, St. Louis, MO, USA) and centrifuged at 10,000 xg at 4°C for 15 min [19]. With the aim of normalization of tissue biochemical results, aliquots of tissue homogenate supernatant were analyzed in duplicate for total protein concentration. The Bio-Rad protein assay kit protocol (Bio-Rad, Mississauga, Canada) was adopted for the determination of lysate protein concentration. The quantitative measurement of the inflammatory marker, TNF-α, (Sigma-Aldrich, St. Louis, MO, USA) and apoptotic marker, caspase-3 (Bio Vision, MA, USA) was done using sandwich ELISA kits according to the manufacturer’s instructions. Tissue results were normalized to total tissue proteins to be expressed as ng/mg protein for TNF-α and pg/mg protein for caspase-3.

### Histopathological Examination

Prefrontal cortical and hippocampal tissue specimens were fixed in 10% buffered formol-saline and processed to get paraffin blocks that were sectioned at 5 µm thick coronal sections, deparaffinized and stained with hematoxylin and eosin (H&E) for histological analysis using light microscopy [32].

### Statistical Analysis

Data were collected and analyzed using SPSS Statistics for Windows (25.0; SPSS Inc., Chicago, IL, USA). Distributions of quantitative variables were tested for normality using Shapiro–Wilk test. If it revealed normal data distribution, parametric tests were applied. If the data were abnormally distributed, non-parametric tests were used. Normally distributed data were expressed as mean and standard deviation. They were analyzed using F-test (one- way ANOVA), followed when significant by Post Hoc test (Tukey) for pair-wise comparisons. Abnormally distributed data were expressed as median and interquartile range. Kruskal Wallis test was used to compare between different groups and pair wise comparison was assessed using Mann–Whitney U test. All tests were two tailed and *p* < 0.05 was considered statistically significant. Graphs were prepared using GraphPad Prism (version 8.0 for Windows).

## Results

### Characterization of the Synthesized SeNPs

**Fig. 2:**
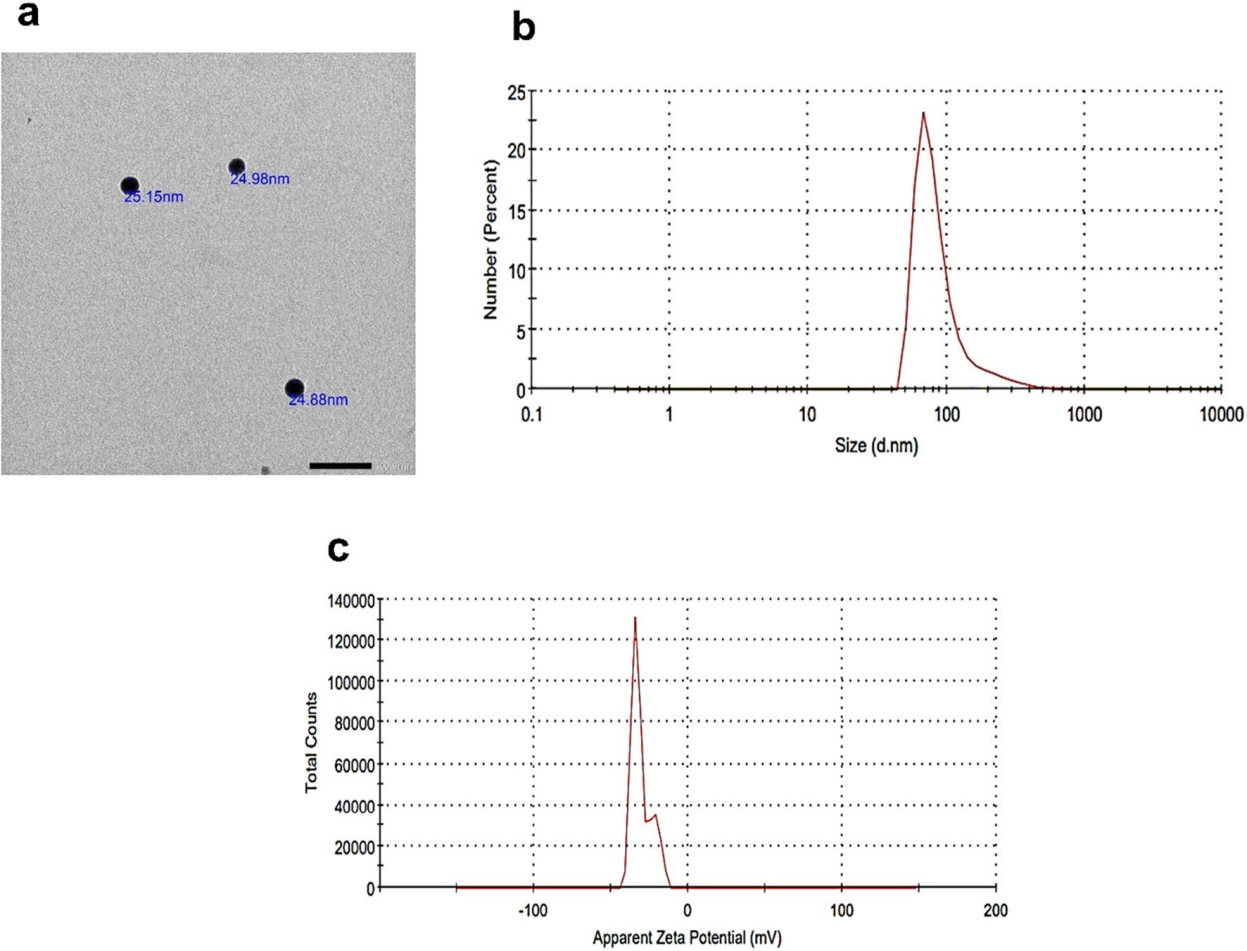
Characterization of SeNPs. Transmission electron micrograph of selenium nanoparticles (scale bar 100 nm) showing size of 20–30 nm in **(a)**. Size distribution of 100% peak at a size of 92.01 nm is shown in **(b)**. Zeta potential of -30.5 mV is revealed in **(c)**

Transmission electron microscope showed that SeNPs were monodispersed spheres, with a size ranging between 20 to 30 nm (Fig. 2a). Dynamic light scattering (DLS) showed a 100% peak at a size of 92.01 nm (Fig. 2b), with a PDI of 0.435. Zeta potential was -30.5 mV (Fig. 2c) and this marks good stability.

### Transmission Electron Microscopic Examination of Prefrontal Cortex and Hippocampus of Rats of SeNPs-treated Group

**Fig. 3:**
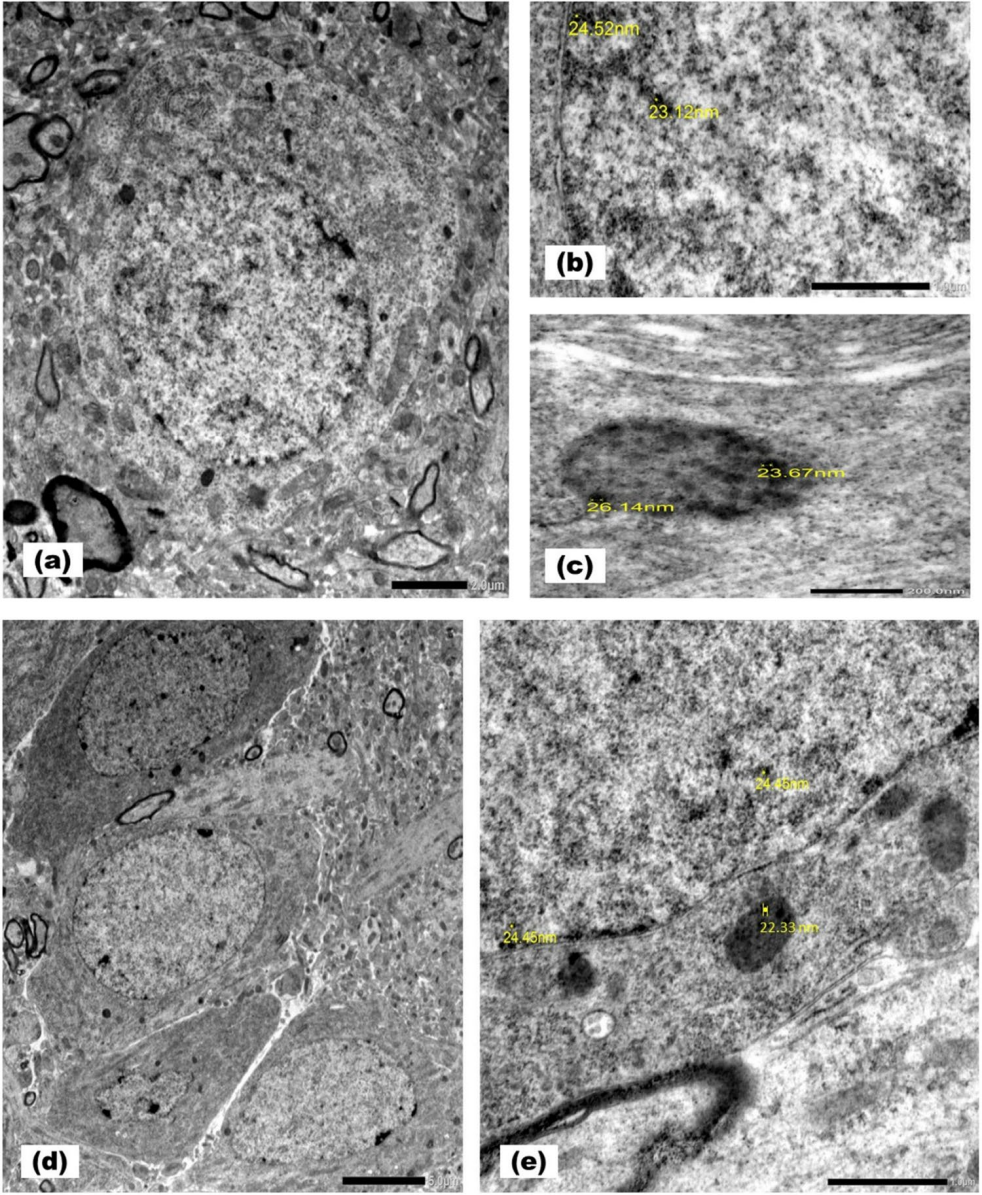
Transmission electron micrographs of prefrontal cortex **a-c** and hippocampus **d-e** of selenium nanoparticles-treated rats. A prefrontal cortical neuron **a** harboring selenium nanoparticles in the nucleus **b** and the lysosomes **c**. Selenium nanoparticles are revealed in the nucleoplasm and lysosomes of a pyramidal neuron in the hippocampus proper **d-e** (Mic. Mag. a × 2500, b × 8000, c × 25000, d × 1200 and e × 8000)

Transmission electron microscopic examination confirmed prefrontal cortical and hippocampal uptake of the different doses of SeNPs used in the present study. Clusters of electron dense SeNPs were seen within the nuclei and the lysosomes of the neuronal perikarya with a size ranging between 20 to 30 nm (Fig. 3a-e).

### Effect of SeNPs on Body Weight

**Fig. 4:**
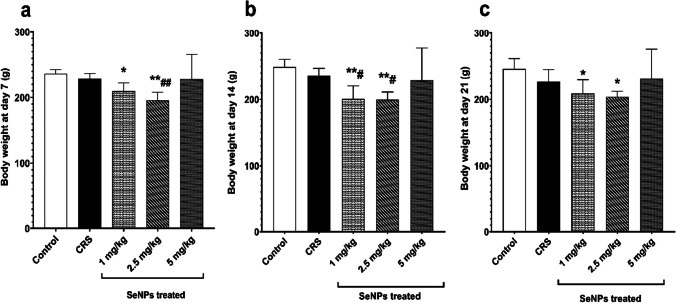
Body weight of rats in the different studied groups at day 7 **(a),** at day 14 **(b)** and at day 21 **(c)**. Data is expressed as Mean ± SD and analyzed by (one-way ANOVA) separately at days 7, 14 and 21, followed when significant by Post Hoc Test (Tukey).* *p* < 0.05, **p ≤ 0.001 significant difference compared to control group. #*p* < 0.05, ##*p* ≤ 0.001 significant difference compared to untreated chronic restraint stress group. CRS: chronic restraint stress, SeNPs: selenium nanoparticles, g: grams

The untreated CRS group did not show any significant change in the body weight of rats at days 7, 14 and 21 of the study, compared to the control group. Treatment of rats with 1 and 2.5 mg/kg SeNPs exhibited a significant reduction in the mean body weight at days 7, 14 and 21 of the study, compared to the control group (Fig. 4a-c). Rats treated with 1 mg/kg SeNPs had a significantly lower body weight at day 14, compared to the untreated CRS rats (Fig. 4b). In addition, rats treated with 2.5 mg/kg SeNPs showed a significant loss of body weight at days 7 and 14, compared with the untreated CRS rats (Fig. 4a-b). Rats treated with 5 mg/kg of SeNPs had no significant difference in the body weight at days 7, 14 and 21 of the study, compared to the control group and untreated CRS group (Fig. 4a-c).

### Effect of SeNPs on Behavioral Tests

1. Elevated plus maze test

**Fig. 5:**
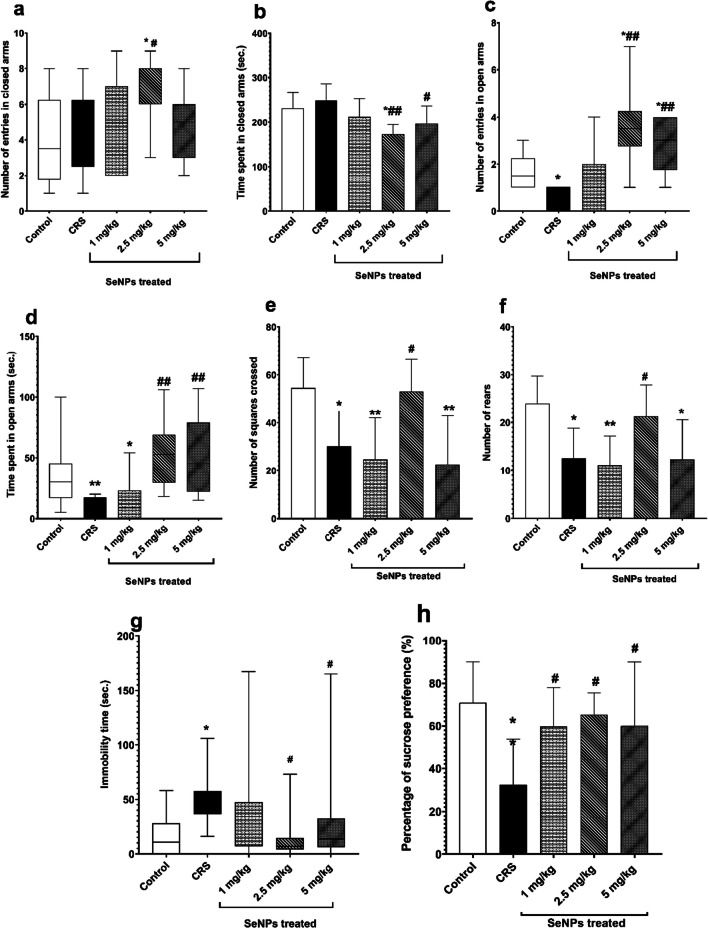
Neurobehavioral parameters of rats in the different studied groups **a-h**. **a** Number of entries in closed arms. **b** Time spent in closed arms. **c** Number of entries in open arms. **d** Time spent in open arms. **e** Number of squares crossed. **f** Number of rears. **g** Immobility time. **h** Percentage of sucrose preference. Data is expressed as median (intra-quartile range) and analyzed by non-parametric ANOVA (Kruskal Wallis, KW) in **a, c, d & g.** Pair wise comparisons are analyzed using Mann Whitney U test. Data is expressed as Mean ± SD and analyzed by (one-way ANOVA), followed when significant by Post Hoc Test (Tukey) in **b, e, f & h.** * *p* < 0.05, **p ≤ 0.001 significant difference compared to control group. #*p* < 0.05**, #**#*p* ≤ 0.001 significant difference compared to untreated chronic restraint stress group. CRS: chronic restraint stress, SeNPs: selenium nanoparticles, sec: seconds

There was no significant difference between the untreated CRS group and the control group regarding number of entries in closed arms and time spent in closed arms (Fig. 5a-b). However, the number of entries in open arms and the time spent in open arms were significantly decreased in the untreated CRS group, versus the control group (Fig. 5c-d).

Treatment of rats with 2.5 mg/kg of SeNPs significantly increased the number of entries in closed arms and decreased the time spent in closed arms versus control group and untreated CRS group. Rats treated with 1 mg/kg of SeNPs did not show significant difference in the number of entries and the time spent in closed arms versus control group and untreated CRS group. Rats treated with 5 mg/kg of SeNPs exhibited significant decrease in the time spent in closed arms versus untreated CRS rats and no significant difference in the number of entries in closed arms versus control group and untreated CRS group (Fig. 5a-b).

The 2.5 and 5 mg/kg doses of SeNPs showed significant rise in the number of entries in open arms and time spent in open arms compared to untreated CRS rats, and caused significantly higher number of entries in open arms than the control group. However, the 1 mg/kg dose of SeNPs did not show significant difference in the number of entries in open arms and time spent in open arms compared to untreated CRS group (Fig. 5c-d).

2. Open field test

Untreated CRS rats showed decreased locomotor activity and anxiety-like behavior, which was evident by significant reduction in both number of squares crossed and number of rears versus the control group. Significant improvement was observed in rats treated with 2.5 mg/kg of SeNPs, where both number of squares crossed and number of rears were significantly increased versus untreated CRS rats. On the contrary, there was no significant improvement observed with rats treated with 1 mg/kg and 5 mg/kg doses of SeNPs compared to untreated CRS rats (Fig. 5e-f).

3. Forced swim test

The immobility time was significantly increased in untreated CRS rats, compared to the control group, indicating behavioral despair. Rats treated with 2.5 mg/kg and 5 mg/kg of SeNPs showed a significant decrease in the immobility time compared to untreated CRS rats. On the other hand, the 1 mg/kg of SeNPs did not show any significant difference in the immobility time compared with untreated CRS group (Fig. 5g).

4. Sucrose preference test

The percentage of sucrose preference in the untreated CRS group was significantly decreased compared with the control group, thus indicating anhedonia. Treatment of rats with all three doses of SeNPs significantly increased the percentage of sucrose preference versus untreated CRS group (Fig. 5h).

### Effect of SeNPs on Neurobiochemical Tests

1. Prefrontal cortical and hippocampal serotonin

**Fig. 6:**
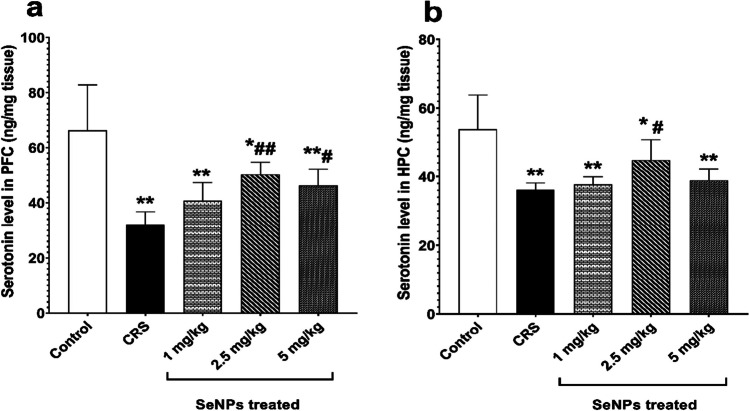
Serotonin level (in ng/mg tissue) in the prefrontal cortex **a** and hippocampus **b** of rats in the different studied groups. Data is expressed as Mean ± SD and analyzed by (one-way ANOVA), followed when significant by Post Hoc Test (Tukey). * *p* < 0.05, **p ≤ 0.001 significant difference compared to control group. #*p* < 0.05, ##*p* ≤ 0.001 significant difference compared to untreated chronic restraint stress group. CRS: chronic restraint stress, SeNPs: selenium nanoparticles, PFC: prefrontal cortex, HPC: hippocampus

Untreated CRS rats showed a significant reduction in the prefrontal cortical tissue levels of serotonin versus the control group. However, treatment of rats with 2.5 mg/kg and 5 mg/kg of SeNPs significantly raised the prefrontal cortical tissue level of serotonin compared to untreated CRS rats. Rats treated with 1 mg/kg of SeNPs did not significantly improve serotonin level compared to untreated CRS rats (Fig. 6a).

In the hippocampal tissues of untreated CRS rats, there was a significant decrease in serotonin level versus the control group. Rats treated with 2.5 mg/kg of SeNPs showed significant rise in serotonin level compared to untreated CRS rats. On the other hand, treatment of rats with either 1 mg/kg or 5 mg/kg of SeNPs did not significantly improve serotonin level compared to untreated CRS rats (Fig. 6b).

2. Oxidative stress markers

**Fig. 7:**
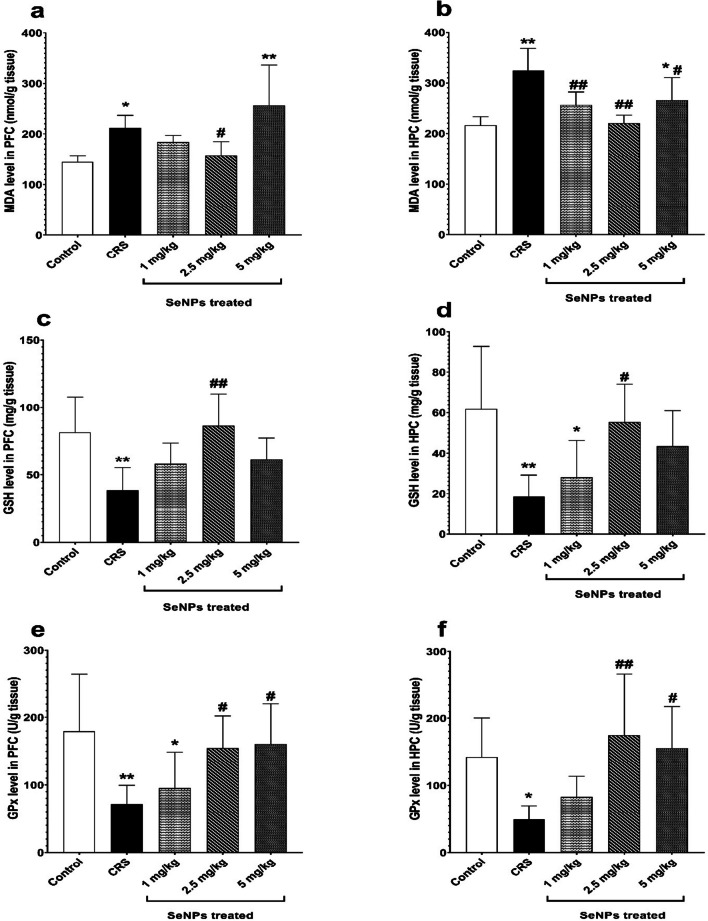
Oxidative stress markers of rats in the different studied groups **(a-f)**. Malondialdehyde level (in nmol/g tissue) in the prefrontal cortex **(a)** and hippocampus **(b)**. Reduced glutathione level (in mg/g tissue) in the prefrontal cortex **(c)** and hippocampus **(d)**. Glutathione peroxidase level (in U/g tissue) in the prefrontal cortex **(e)** and hippocampus **(f)**. Data is expressed as Mean ± SD and analyzed by (one-way ANOVA), followed when significant by Post Hoc Test (Tukey). * *p* < 0.05, **p ≤ 0.001 significant difference compared to control group. #*p* < 0.05, ##*p* ≤ 0.001 significant difference compared to untreated chronic restraint stress group. CRS: chronic restraint stress, SeNPs: selenium nanoparticles, MDA: malondialdehyde, GSH: reduced glutathione, GPx: glutathione peroxidase, PFC: prefrontal cortex, HPC: hippocampus

In the prefrontal cortical tissues of untreated CRS rats, there was a significant increase in MDA levels versus control group, indicating lipid peroxidation. Rats treated with 2.5 mg/kg of SeNPs had a significant reduction in the prefrontal cortical tissue level of MDA versus untreated CRS rats. However, treatment of rats with either 1 mg/kg or 5 mg/kg of SeNPs did not significantly decrease MDA levels compared to untreated CRS group (Fig. 7a). In the hippocampal tissues of untreated CRS rats, there was a significant elevation in MDA level, compared to the control group, indicating lipid peroxidation. Treatment with all three doses of SeNPs significantly reduced the MDA levels compared to untreated CRS rats (Fig. 7b).

In the prefrontal cortical tissue of untreated CRS rats, there was a significant reduction in GSH levels versus the control group. Treatment of rats with 2.5 mg/kg of SeNPs caused a significant rise in the prefrontal cortical tissue level of GSH versus untreated CRS rats. On the contrary, treatment of rats with either 1 mg/kg or 5 mg/kg of SeNPs did not result in significant elevation in the GSH levels compared to untreated CRS group (Fig. 7c). Regarding the hippocampal tissue levels of GSH, it was significantly decreased in untreated CRS rats versus the control group. Rats treated with 2.5 mg/kg of SeNPs exhibited a significant rise in GSH level versus untreated CRS rats. However, rats treated with 1 mg/kg and 5 mg/kg doses of SeNPs did not cause significant elevation in the GSH levels compared to untreated CRS group (Fig. 7d).

Untreated CRS rats showed a significant reduction in glutathione peroxidase activity in the prefrontal cortical tissue, compared to the control group. Rats treated with 2.5 mg/kg and 5 mg/kg of SeNPs showed a significant improvement in glutathione peroxidase activity in the prefrontal cortical tissue compared to untreated CRS rats. On the other hand, rats treated with 1 mg/kg of SeNPs did not exhibit any significant improvement compared to untreated CRS group (Fig. 7e). In the hippocampal tissues of untreated CRS rats, there was a significant reduction in glutathione peroxidase activity, versus the control group. Treatment of rats with 2.5 mg/kg and 5 mg/kg of SeNPs caused a significant elevation in glutathione peroxidase activity versus untreated CRS rats. On the other hand, rats treated with 1 mg/kg of SeNPs did not cause any significant improvement versus untreated CRS group (Fig. 7f).

3. Prefrontal cortical and hippocampal tumor necrosis factor-alpha (TNF-α)

**Fig. 8:**
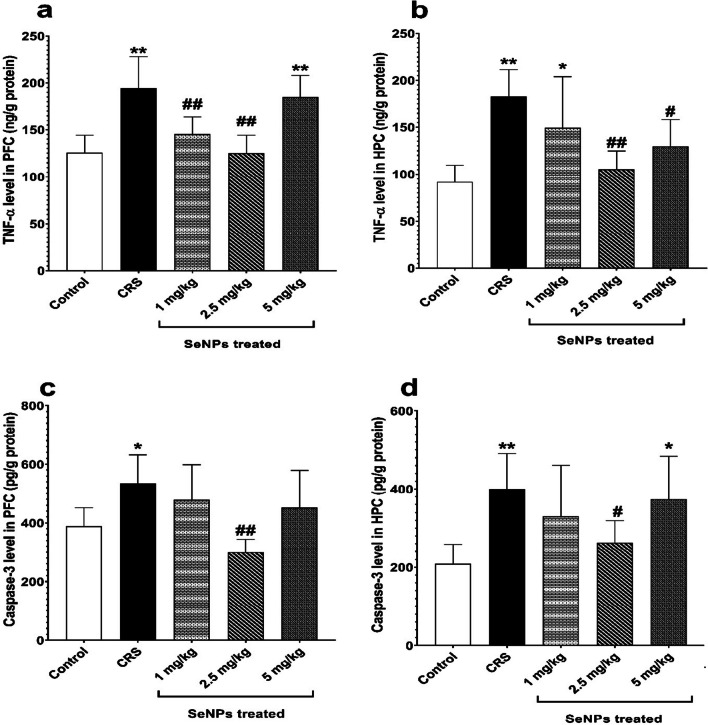
TNF-α level (in ng/g protein) in the prefrontal cortex **a** and hippocampus **b**, and caspase-3 level (in pg/g protein) in the prefrontal cortex **c** and hippocampus **d** of rats in the different studied groups. Data is expressed as Mean ± SD and analyzed by (one-way ANOVA), followed when significant by Post Hoc Test (Tukey). * *p* < 0.05, **p ≤ 0.001 significant difference compared to control group. #*p* < 0.05, ##*p* ≤ 0.001 significant difference compared to untreated chronic restraint stress group. CRS: chronic restraint stress, SeNPs: selenium nanoparticles, TNF-α: tumor necrosis factor alpha, PFC: prefrontal cortex, HPC: hippocampus

In untreated CRS rats, there was a significant increase in the prefrontal cortical tissue levels of TNF-α versus the control group, indicating neuroinflammation. Rats treated with 1 mg/kg and 2.5 mg/kg of SeNPs showed a significant reduction in the prefrontal cortical tissue level of TNF-α versus untreated CRS group. On the contrary, rats treated with 5 mg/kg of SeNPs did not significantly improve the TNF-α levels compared to untreated CRS rats (Fig. 8a).

Regarding the hippocampal tissue levels of TNF-α, it was significantly increased in untreated CRS rats versus the control group, indicating neuroinflammation. Treatment with 2.5 mg/kg and 5 mg/kg of SeNPs caused a significant decrease in TNF-α level versus untreated CRS group. However, treatment with 1 mg/kg of SeNPs did not significantly improve the TNF-α levels compared to untreated CRS group (Fig. 8b).

4. Prefrontal cortical and hippocampal caspase-3

Untreated CRS rats showed a significant increase in the prefrontal cortical tissue levels of caspase-3 versus the control group, indicating apoptosis. Treatment of rats with 2.5 mg/kg of SeNPs significantly decreased the prefrontal cortical tissue level of caspase-3 versus untreated CRS rats. However, treatment of rats with either 1 mg/kg or 5 mg/kg of SeNPs did not significantly improve caspase-3 levels compared to untreated CRS rats (Fig. 8c).

Regarding the hippocampal tissues, untreated CRS rats showed a significant elevation in caspase-3 level, compared to the control group, indicating apoptosis. Treatment of rats with 2.5 mg/kg of SeNPs significantly reduced caspase-3 level versus untreated CRS group. On the other hand, treatment of rats with either 1 mg/kg or 5 mg/kg of SeNPs did not significantly improve caspase-3 levels compared to untreated CRS group (Fig. 8d).

### Effect of SeNPs on Histopathological Examination

1. Light microscopic examination of prefrontal cortices

**Fig. 9:**
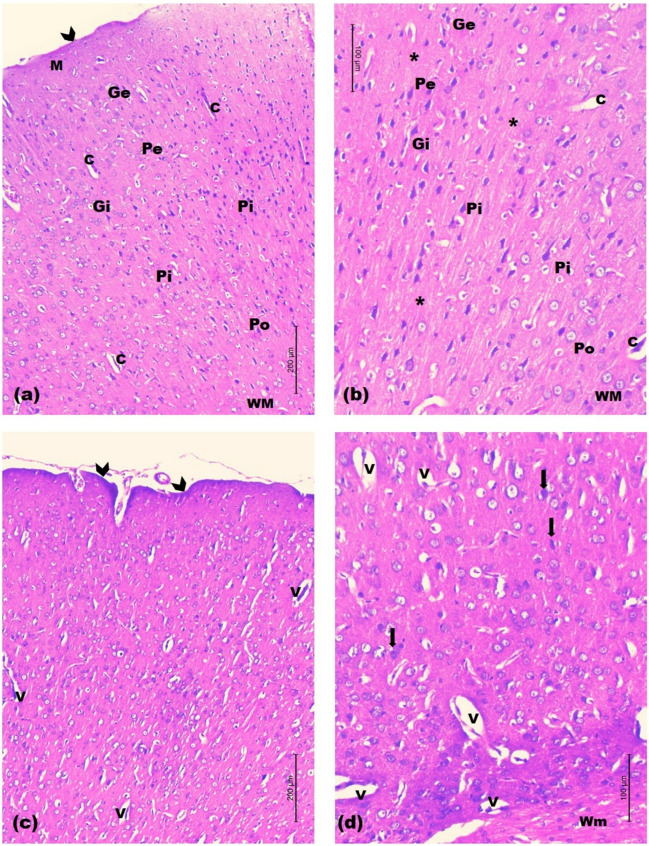
Light H&E-stained micrographs of the prefrontal cortices of control and untreated chronic restraint stress rats. Histological sections of control rats in **(a)** and **(b)**. The normal architecture with the molecular layer (M) beneath the pia mater (arrow head) is seen. The external granular (Ge), the external pyramidal (Pe) and the internal granular (Gi) layers are observed. The internal pyramidal cell layer (Pi) is seen with its large pyramidal neuronal cell bodies. The polymorphic layer of the cortex (Po) is seen deeper to the internal pyramidal cell layer. The neuropil (asterisk) is revealed among the cell layers with numerous blood capillaries (C). White matter (WM). Histological sections of untreated chronic restraint stress rats in **(c)** and **(d)**. The irregular cortical surface is seen (arrow heads). The disturbed neuronal layers are seen within a lightly-stained spongy-like neuropil with vacuoles (V). Occasional pyknotic nuclei are seen (arrows). Notice, the disorganized white matter (Wm) (Mic. Mag. a × 100, b × 200, c × 100 and d × 200)

In control rats, examination of prefrontal cortex showed normal laminated appearance with six different layers of variable thickness. The layers were from superficial to deep; the molecular layer, beneath the pia mater, composed mainly of fibers and few nerves cell bodies; the external and internal granular layers showed the same architecture, they were formed of small, densely packed stellate neurons (granule cells); the external pyramidal layer consisted of small and medium-sized pyramidal neurons. The internal pyramidal layer was characterized by the prominent large and medium-sized pyramidal neurons, that have triangular cell bodies and large euchromatic nuclei. The deepest cortical layer, the polymorphic cell layer composed of loosely arranged cells of varying shapes and sizes. Deeper to it, the white matter was seen as regularly arranged bundles of nerve fibers separated by rows of neuroglia. The cortical neuropil was revealed as amorphous pale eosinophilic background among neuroglia and neuronal cell bodies (Fig. 9a-b).

In untreated CRS rats, the prefrontal cortical sections exhibited various degrees of degenerative changes that involved the neuronal cell layers and the white matter. Irregularity of the cortical surface was observed. The well-defined laminated pattern was almost lost with evident neuronal loss. Many neurons in different cortical layers appeared distorted, compared to those of control sections. They were surrounded by a lightly-stained spongy-like neuropil with many mouth-eaten small and large vacuoles. Occasional pyknotic nuclei of neurons were seen. The white matter showed several vacuoles between the disorganized bundles of nerve fibers (Fig. 9c-d).

**Fig. 10:**
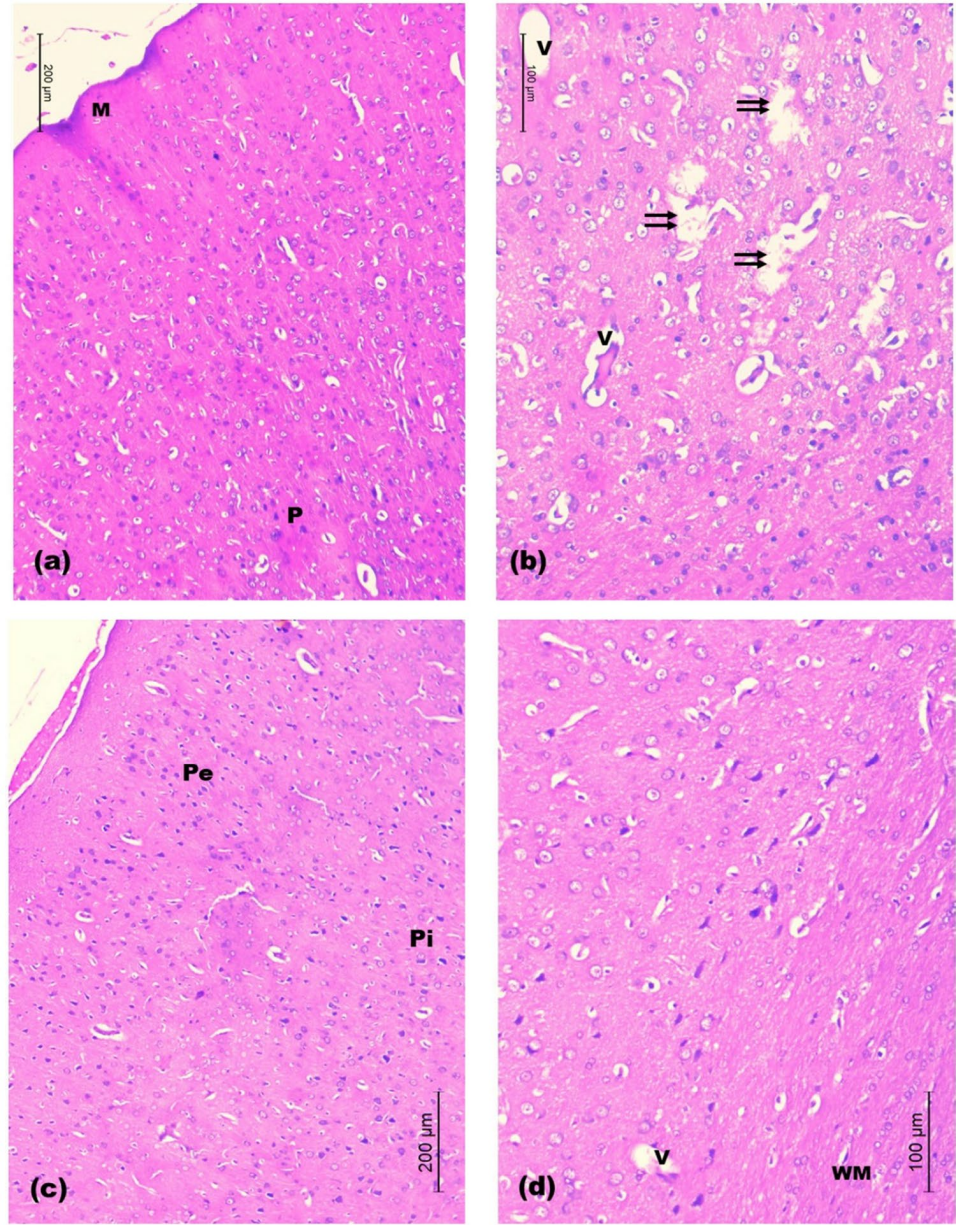
Light H&E-stained micrographs of the prefrontal cortices of rats treated with 1 and 2.5 mg/kg of SeNPs. Sections of rats treated with 1 mg/kg SeNPs in **a** and **b** show restoration of the laminar architecture of cortical neurons (M, P). Few vacuoles (V) are seen with sporadic areas of mouth-eaten neuropil (double arrows). Sections of rats treated with 2.5 mg/kg SeNPs in **c** and **d** reveal the normal architecture of cortical neurons and the neuropil. Few small vacuoles are seen (V). Note, the regularly arranged nerve fiber bundles of the white matter (WM). External (Pe) and internal pyramidal (Pi) cell layers (Mic. Mag. a × 100, b × 200, c × 100 and d × 200)

In rats treated with 1 mg/kg SeNPs, the prefrontal cortical sections almost resembled those of the untreated CRS group except that the cortical surface was almost regular and the cortical neurons in various layers showed their normal morphology and laminar pattern. Manifestations of localized spongy-like neuropil were revealed with few vacuoles and sporadic mouth-eaten areas (Fig. 10a-b).

In rats treated with 2.5 mg/kg SeNPs, the prefrontal cortical sections showed that the appearance of prefrontal cortex was almost comparable to the control sections. Normal laminar pattern was revealed with prominent pyramidal cell layers. The smooth cortical surface was seen with nearly normal appearance of the neuropil. Normal arrangement of nerve fiber bundles of the white matter with nuclei of neuroglia was seen in between. Occasionally few vacuoles were depicted; however, they were small and localized (Fig. 10c-d).

**Fig. 11:**
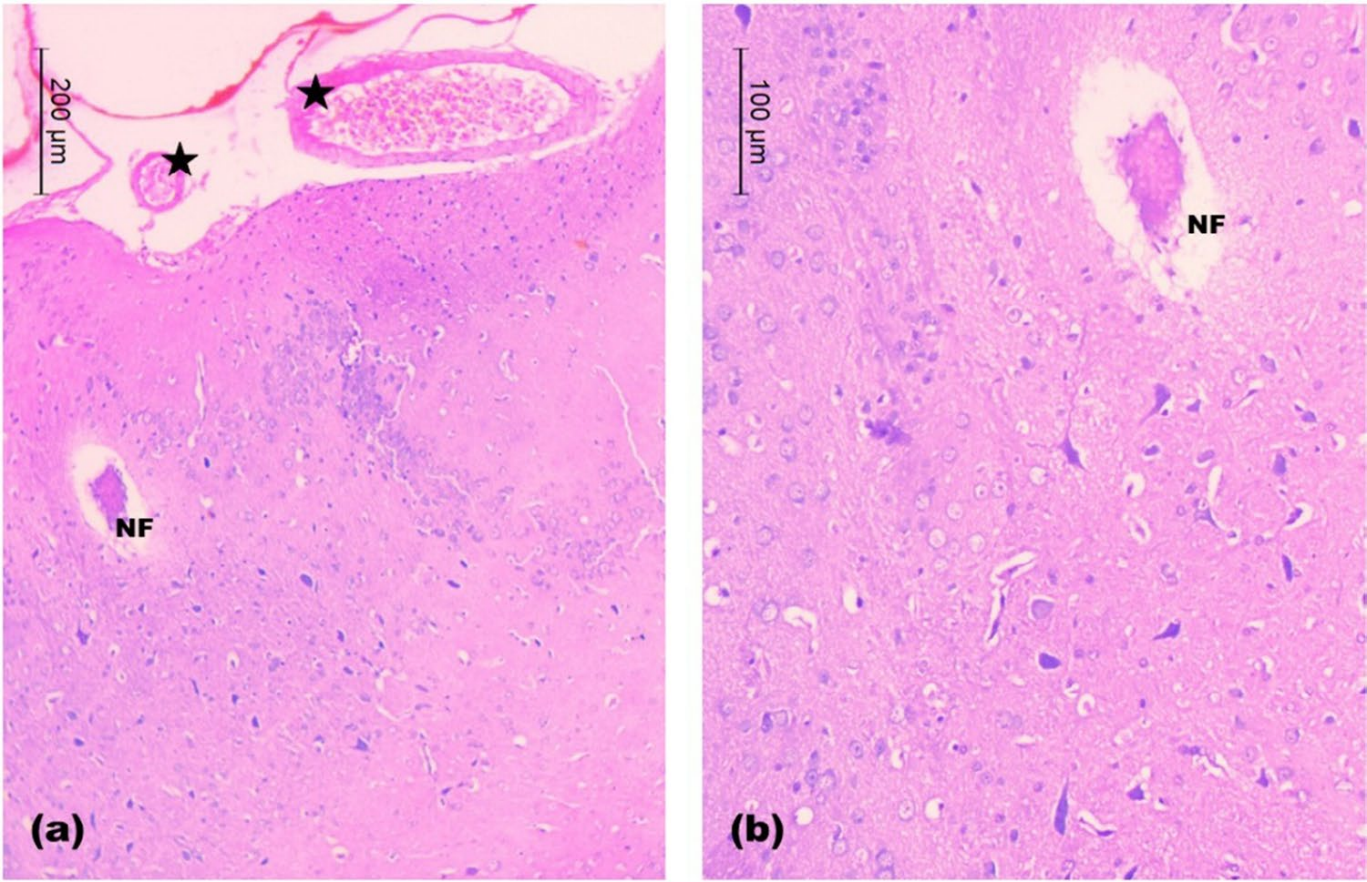
Light H&E-stained micrographs of the prefrontal cortices of rats treated with 5 mg/kg of SeNPs in **a** and **b** show congested blood vessels of the subarachnoid space (stars). Vacuolar necrotic areas are revealed with structureless fibrillar material (NF). Note, disorganized cortical layers (Mic. Mag. a × 100, b × 200)

In rats treated with 5 mg/kg SeNPs, examination of prefrontal cortex of rats revealed irregularity of the cortical surface with congested large blood vessels of the subarachnoid space. Disorganization of most cortical layers was depicted. Vacuolar necrotic areas were seen containing structureless fibrillar material (Fig. 11a-b).

2. Light microscopic examination of the hippocampi

**Fig. 12:**
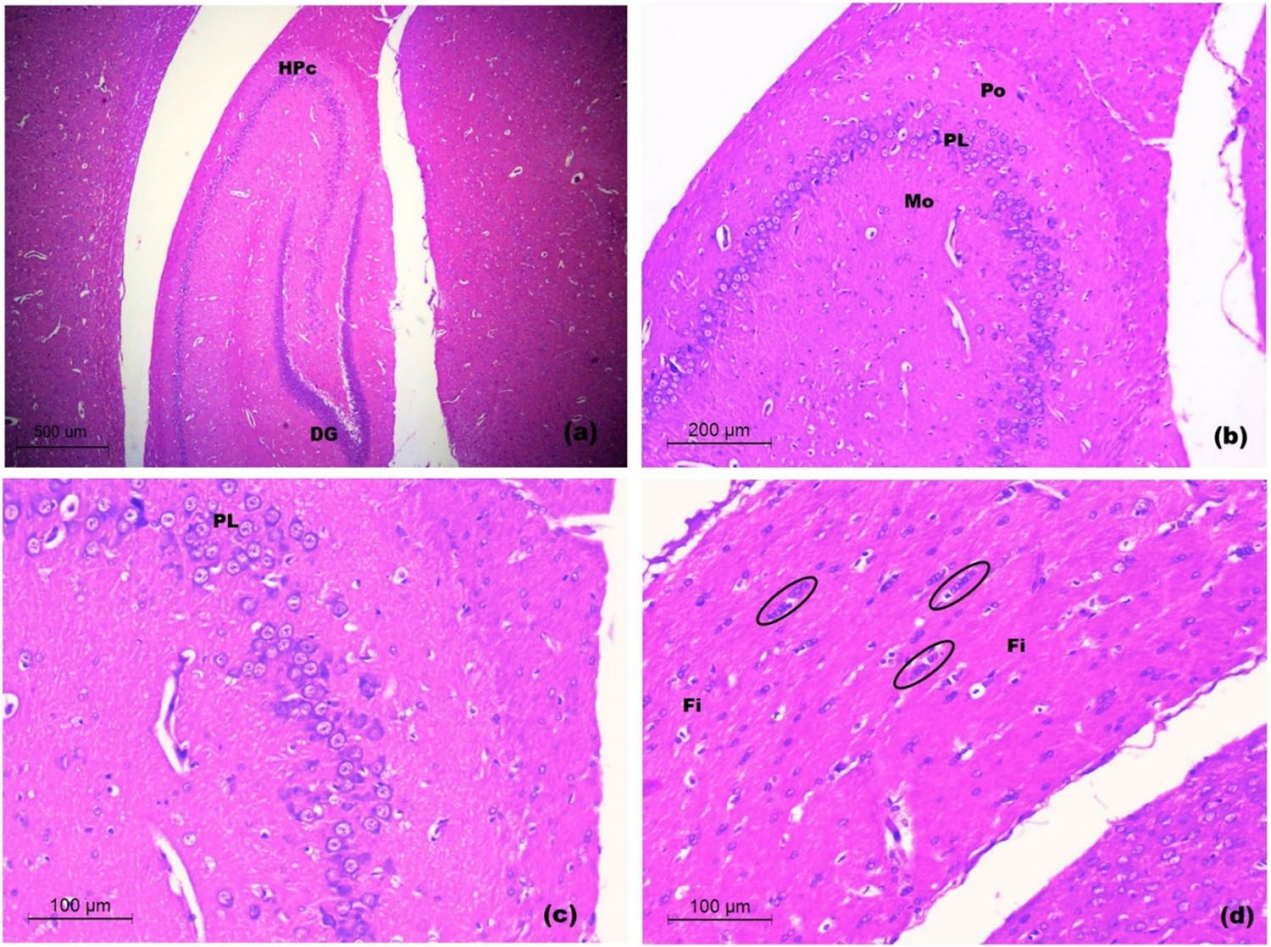
Light H&E-stained micrographs of hippocampus of control rats. Sections of control rats in **a-d**. The normal architecture of hippocampus proper (HPc) and dentate gurus (DG) is seen. The hippocampus proper appears with the molecular layer (Mo), the prominent pyramidal cell layer (PL) and the polymorphic layer (Po). Note, the well-organized fimbria (Fi) and the rows of neuroglia (Oval) (Mic. Mag. a × 40, b × 100, c × 200 and d × 200)

In control rats, examination of hippocampus showed normal architecture of hippocampus proper (cornu-ammonis). Three distinct layers were revealed; the innermost molecular cell layer containing nerve cell processes and few neurons. A middle pyramidal cell layer containing rows of hippocampal pyramidal cells with large euchromatic nuclei and triangular cell bodies. The outer polymorphic layer was also revealed. Fimbria showed properly arranged nerve fiber bundles separated by blood capillaries and rows of neuroglia (Fig. 12a-d).

**Fig. 13:**
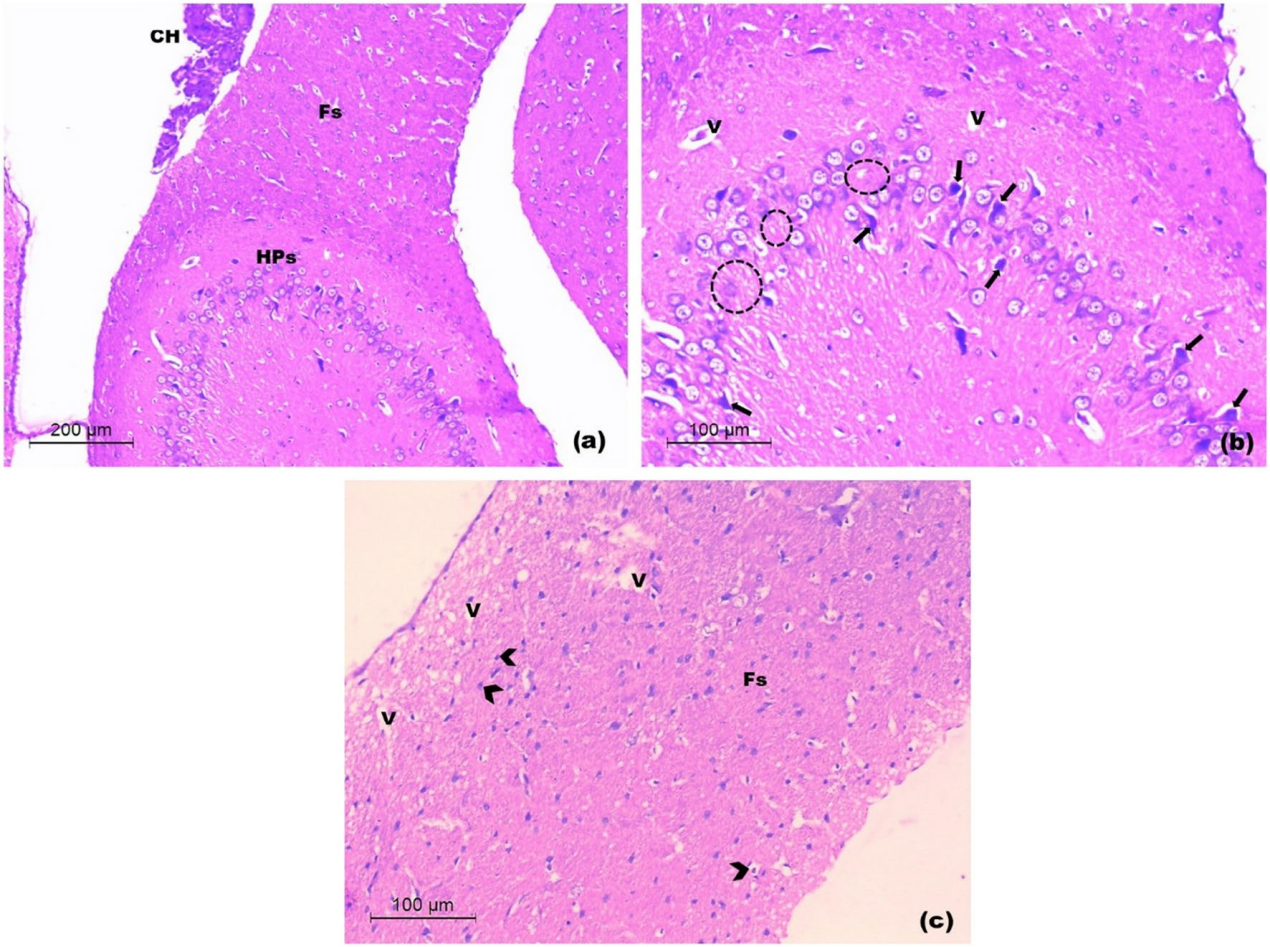
Light H&E-stained micrographs of hippocampus untreated CRS rats **a-c**. The disorganized pyramidal layer of hippocampus proper (HPs in a) is seen with evident neuronal loss (dotted circles in b) and focal vacuoles of the polymorphic layer (V in b). Many apoptotic pyramidal neurons are revealed (arrows in b). Note, the disorganized fimbria (Fs in a and c) with vacuoles (V in c) and cellular infiltration (arrow heads in c). The choroid plexus of inferior horn of lateral ventricle (CH in a) is seen (Mic. Mag. a × 100, b × 200 and c × 200)

In untreated CRS rats, the hippocampal sections exhibited disorganization of the pyramidal cell layer of hippocampus proper with evident neuronal loss. Many apoptotic pyramidal neurons appeared with shrunken cell bodies, hyper eosinophilic cytoplasm and pyknotic nuclei. Some vacuoles were seen in the polymorphic layer. Areas of dissolution of the white matter in fimbria were also revealed with breakup of its nerve fiber bundles, accompanied by vacuolation. Limited areas of cellular infiltration were seen (Fig. 13a-c).

**Fig. 14:**
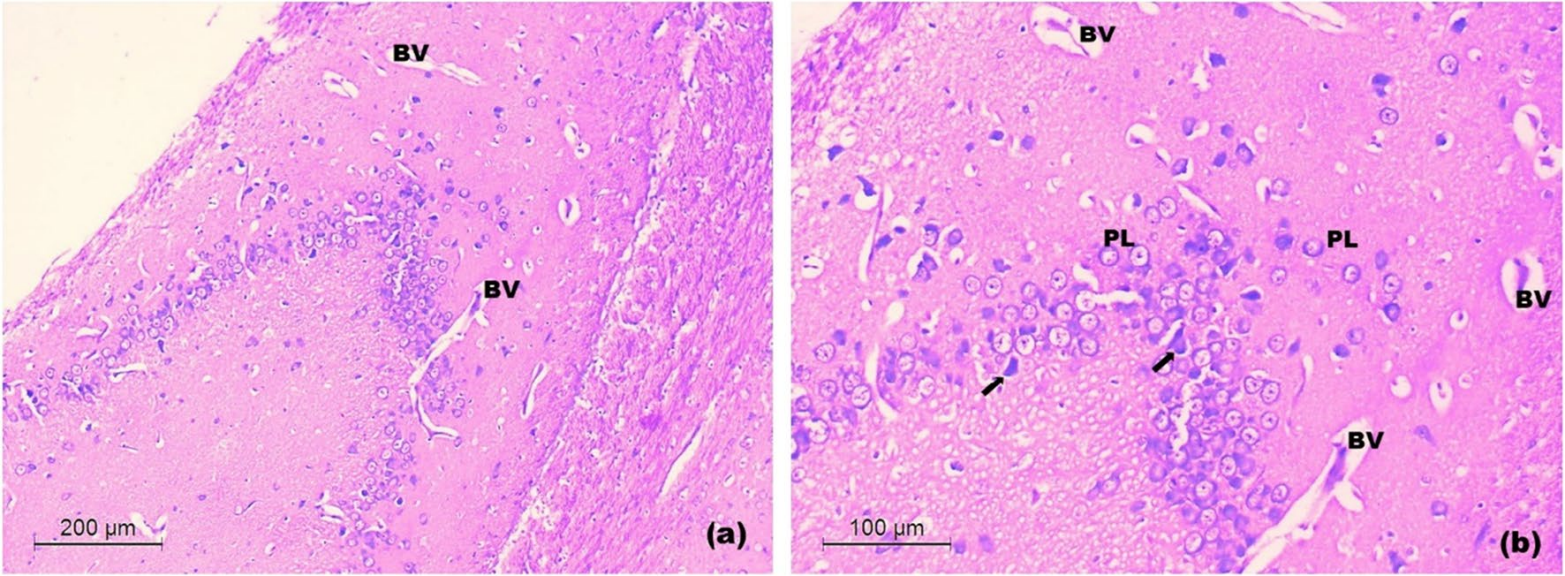
Light H&E-stained micrographs of hippocampus of rats treated with 1 and 2.5 mg/kg of SeNPs. Sections **a** and **b** show the almost normal architecture of hippocampus proper and fimbria with prominent capillaries and larger blood vessels (BV). A number of pyramidal neurons (PL) are seen extending into the polymorphic layer. Note, very few apoptotic pyramidal neurons (arrows) (Mic. Mag. a × 100, b × 200)

In rats treated with 1 mg/kg and 2.5 mg/kg doses of SeNPs, examination of hippocampal sections gave similar histological results. They almost exhibited the normal organization of hippocampus proper and fimbria with evident increased vascularity in the form of numerous capillaries and large-sized blood vessels. The pyramidal cell layer of hippocampus proper depicted relative hypercellularity with variable-sized neurons extending into the polymorphic layer. Sporadic apoptotic pyramidal neurons were seen with shrunken cell bodies, hyper eosinophilic cytoplasm and pyknotic nuclei (Fig. 14a-b).

**Fig. 15:**
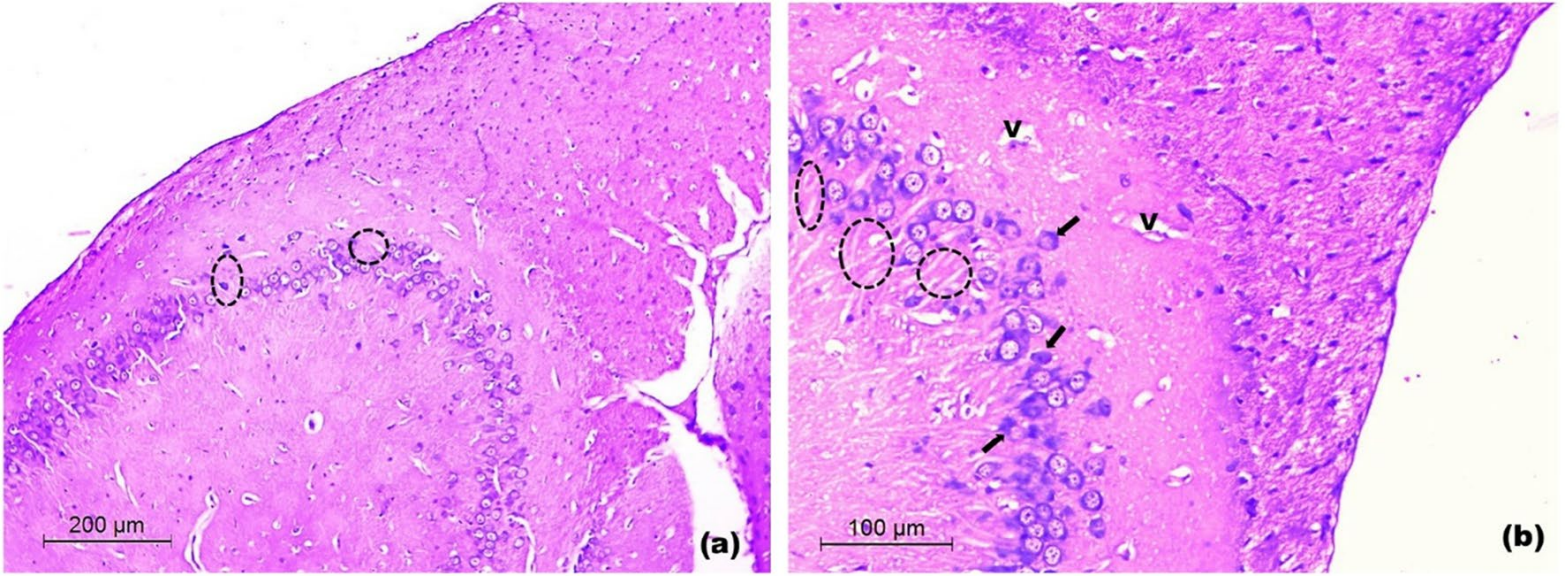
Light H&E-stained micrographs of hippocampus of rats treated with 5 mg/kg of SeNPs **a-b**. Evident thinning out of the pyramidal cell layer with neuronal loss (dotted circles in a and b) is seen. Some apoptotic pyramidal cells (arrows in b). Note, few vacuoles (V in b) of the polymorphic layer of hippocampus proper (Mic. Mag. a × 100, b × 200)

In rats treated with 5 mg/kg SeNPs, examination of hippocampus revealed no apparent increased vascularity. Areas of neuronal loss with decreased thickness of the pyramidal cell layer of hippocampus proper were depicted. Some neurons appeared with dark shrunken cell bodies. The fimbria was apparently normal (Fig. 15a-b).

## Discussion

In the present study, we examined the protective effects of different doses of SeNPs on the body weight, neurobehavioral scores, biochemical and histological parameters of the prefrontal cortex and hippocampus in a model of CRS in rats. Treatment with different doses of SeNPs demonstrated variable effectiveness in ameliorating these changes, with the 2.5 mg/kg dose showing the best improving results in all studied parameters. To the best of our knowledge, this is the first study to explore the potential protective role of SeNPs in CRS.

Our study proved the uptake of different doses of SeNPs by the neurons of the prefrontal cortex and hippocampus using TEM. Characterization of SeNPs revealed that their size ranged from 20–30 nm, which contributed to the ability of SeNPs to cross the blood brain barrier. In addition, the glucose coating used while synthesizing SeNPs allowed their delivery into the neurons through interacting with the glucose transporters present in the membrane of the endothelial cells of the blood brain barrier.

Regarding body weight, CRS did not cause any change in body weight of rats on days 7, 14 and 21 of the present study compared to the control group. However, previous studies reported decrease in body weight of rats exposed to CRS in comparison to control rats [33–48]. Such contradiction between our study and other studies could be because the 21-day stress paradigm used in our study could not cause profound hypothalamic damage that may become evident with longer stress durations. Furthermore, Wistar rats used in our study may differ in their response to food intake and body weight when exposed to CRS with other rat strains, such as Sprague–Dawley rats which were also used in some of the previous studies. Only the rats treated with 1 mg/kg and 2.5 mg/kg doses of SeNPs showed significant reduction in the body weight at days 7, 14 and 21 of the study, versus the control group. Al-Quraishy et al. also found that SeNPs reduced body weight in streptozotocin-induced diabetic rats and denoted such finding to the ability of SeNPs to decrease food intake [49]. It was reported that selenoproteins can increase hypothalamic response to leptin and accordingly reduce food intake [50].

The present study showed that CRS induced oxidative stress through increasing MDA level and decreasing the level of reduced glutathione and glutathione peroxidase activity in the prefrontal cortical and hippocampal tissues versus control. These were supported by Ning et al. [21], Grundmann et al. [46], Fontella et al. [51] and Lin et al. [52]. This most likely occurred because chronic stress led to excessive production of glucocorticoids, which bind to their receptors in the prefrontal cortex and hippocampus inducing oxidative stress state by two mechanisms. The first one is through increasing mitochondrial oxidative phosphorylation, which in turn increased ROS generation and lipid peroxidation. The second one is abolishing the antioxidant defense response through decreasing the level of endogenous antioxidants [53]. In addition, CRS resulted in neuroinflammation and apoptosis and this was evidenced by elevating the levels of prefrontal cortical and hippocampal TNF-α and caspase-3 versus control. Jangra et al. [23], Banagozoar Mohammadi et al. [54], Novaes et al. [55], Pezeshki-Nia et al. [56], and Nouri et al. [57] supported CRS-induced neuroinflammation. While Kuswati [58], Orlovsky et al. [59] and Seo et al. [60] supported CRS-induced neuronal apoptosis. CRS-induced neuroinflammation could be explained by brain microglial activation, which led to increased production of pro-inflammatory cytokines. The high level of glucocorticoids released during chronic stress directly activates microglia of the prefrontal cortex and hippocampus since they express both glucocorticoid receptor and mineralocorticoid receptor. Chronic stress was also evidenced to cause peripheral low grade-inflammation due to the development of a state of glucocorticoid resistance, which eventually decreases the number of glucocorticoid receptors in the cytoplasm and downregulates glucocorticoid receptor driven anti-inflammatory genes. This causes the immune cells in the periphery to secrete large quantities of pro-inflammatory markers into the circulation, which cross the blood brain barrier and activate microglia. Furthermore, the pro-inflammatory cytokines directly stimulate HPA axis [61, 62]. Moreover, the brain oxidative stress state leads to dysregulation of the signaling pathways that modulate the immunological response within the CNS, with resultant upregulation of signaling pathways and transcription factors, such as phosphoinositide 3-kinase (PI3K)/Akt, MAPK and NF-κB, which eventually promote pro-inflammation [63, 64]. CRS-induced neuronal apoptosis could be explained by the activation of both intrinsic and extrinsic apoptotic pathways because of neuronal oxidative stress and neuroinflammation [65]. Chronic stress-induced glucocorticoids secretion was also shown to increase pro-apoptotic BAX protein expression and decrease anti-apoptotic Bcl-2 expression. As a result, BAX becomes inserted into the mitochondrial membrane leading to the release of cytochrome C, which binds the apoptotic activating factor-1 in the cytoplasm forming apoptosome. Then, the apoptosome activates caspase-9, which in turn activates caspase-3 [58, 66]. On the other hand, the 2.5 mg/kg dose of SeNPs was highly effective in preventing the neuronal oxidative stress, neuroinflammation and apoptosis induced by CRS. Previous studies noted the mitigating effect of SeNPs on the brain oxidative stress both in vivo [67–73] and in vitro [74, 75]. About 25 selenoproteins are expressed in the brain and they are abundant in the hippocampus and cerebral cortex [76]. SeNPs exert their antioxidant activity through incorporation into selenoproteins. Some of these selenoproteins are enzymes, known as selenoenzymes, that are needed for scavenging ROS. Of these selenoenzymes is the glutathione peroxidase, which removes the hydrogen peroxides by oxidizing glutathione [17]. Glutathione peroxidase isoenzyme 1 (GPX 1) and isoenzyme 4 (GPX4) are present at high levels in the brain [76]. SeNPs increase both the level and activity of these selenoenzymes. They have been shown to eradicate several ROS, such as superoxide anion, 1,1-diphenyl-2-picrylhydrazyl, singlet oxygen and carbon-centered free radicals [77]. Many studies demonstrated the alleviating effect of SeNPs on neuroinflammation [68, 70, 71]. The anti-inflammatory action of SeNPs can be explained by their ability to downregulate inflammatory transcription factors, such as NF-κB and MAPK and their signaling pathways [17], thus inhibiting microglial release of pro-inflammatory cytokines. In addition, selenoprotein S (SELENOS), selenoprotein K (SELENOK), selenoprotein P (SELENOP) and selenoprotein R (SELENOR) are involved in the regulation of neuroinflammation through decreasing the release of proinflammatory cytokines from microglia, increasing phagocytosis of microglia and decreasing microglial proliferation. Thus, SeNPs can exert their anti-inflammatory action through upregulation of these selenoproteins [76]. Yuan et al. [70] and Bashir et al. [67] also agreed with the anti-apoptotic activity of SeNPs on neurons through denoting their ability to elevate Bcl-2 expression and lower BAX expression. Accordingly, SeNPs can deactivate apoptotic pathways through their antioxidant and anti-inflammatory effects.

Our study also noted decreased prefrontal cortical and hippocampal serotonin levels in the untreated CRS group versus the control group. Similar results were obtained by Wang et al. [78], Aboul-Fotouh [34], Liang et al. [35] and Sunanda et al. [79]. This can be attributed to the reduced firing rate of the serotonergic neurons that occur as a result of the excessive generation of glucocorticoids by chronic stress. Several research studies also showed that chronic stress causes the downregulation of 5-hydroxytryptamine 1A (5-HT_1A_) receptor in both prefrontal cortex and hippocampus [80]. In addition, neuroinflammation was reported to induce tryptophan metabolism through the kynurenine pathway by the enzyme indoleamine-2,3-dioxygenase. Consequently, this lowers the influx of tryptophan into the brain, hence decreasing serotonin synthesis [81, 82]. Moreover, increased ROS generation renders the enzyme tryptophan hydroxylase in an oxidizing state that in turn deactivates its catalytic function and thus, serotonin synthesis is abolished [83]. Treatment with the 2.5 mg/kg dose of SeNPs significantly attenuated the decrease in both prefrontal cortical and hippocampal serotonin levels versus untreated CRS group. Al Kahtani [72] and Abou Zaid et al. [84] also noted elevated brain serotonin levels by SeNPs in rat models of cadmium and acrylamide-induced neurotoxicity respectively. They supported such finding by the antioxidant capacity of SeNPs. Thus, our study proposes that anti-inflammatory and anti-apoptotic actions of SeNPs, in addition to their antioxidant activity, to be the explanation for the restored levels of prefrontal cortical and hippocampal serotonin.

Regarding the behavioral tests, CRS induced anxiety-like behavior, through reducing number of entries in the open arms and less time spent in open arms in the elevated plus maze test and decreasing number of rears in the open field test versus control group. CRS also induced depressive-like behavior by increasing immobility time in the forced swim test and decreasing percentage of sucrose consumption in the sucrose preference test versus control group. In addition, CRS decreased locomotor activity through decreasing number of squares crossed in the open field test, compared to the control group. Peng et al. [44], Luo et al. [43], Bagheri et al. [39], Zhu et al. [85], Carneiro de Oliveira et al. [86], Nade et al. [20], Sheng et al. [87], Shen et al. [88] and Lee et al. [89] reported similar results. These neurobehavioral changes could be explained by the decreased prefrontal cortical and hippocampal serotonin levels [35]. Also, increased prefrontal cortical and hippocampal oxidative stress, TNF-α levels and caspase-3 levels depicted in our study upon exposure to chronic stress definitely contribute to these behavioral alterations [54]. SeNPs treatment significantly alleviated anxiety-like and depressive-like behaviors, specifically the 2.5 mg/kg dose, versus untreated CRS group. Ebokaiwe et al. [69] and Khalil et al. [90] reported improvement of several neurobehavioral parameters by SeNPs in rat models of streptozotocin-induced diabetes and deltamethrin-induced neurotoxicity respectively. Thus, SeNPs improved the neurobehavioral indices through restoring the prefrontal cortical and hippocampal serotonin levels, their antioxidant, anti-inflammatory and anti-apoptotic actions.

In the histological assessment, CRS exhibited evident neuronal loss and appearance of apoptotic pyramidal neurons with pyknotic nuclei in the prefrontal cortex and hippocampus. Such findings were supported by Salama et al. [91], Jayakumar et al. [92], Feng et al. [93], Chandrasekhar et al. [94], Becerril-Chavez et al. [1] and de Lima et al. [95]. They can be explained by the occurrence of reactive gliosis in the prefrontal cortex and hippocampus in response to neuroinflammation [95], which is evidenced by high prefrontal cortical and hippocampal TNF-α levels in our study. Also, elevated level of prefrontal cortical and hippocampal caspase-3 contributes to these findings. We also noted that the damage depicted by CRS in the prefrontal cortex was far more extensive and profound than that in the hippocampus. This may be supported by the prefrontal cortex being a highly organized structure [96], while the hippocampus is considered as a primitive cortical tissue [97]. Thus, on the structural level, the prefrontal cortical tissue is more sensitive to chronic stress than the hippocampal tissue. Only the 2.5 mg/kg dose of SeNPs was able to restore the normal architecture of the prefrontal cortex. Both 1 mg/kg and 2.5 mg/kg doses of SeNPs preserved the normal organization of the hippocampus through inducing hypervascularity and relative hypercellularity in the pyramidal cell layer. Abozaid noted that SeNPs slightly increased vascularity of the hippocampus proper in rat model of acrylamide-induced neurotoxicity [98]. Ibrahim et al. illustrated that SeNPs increased thickness of pyramidal cell layer of hippocampus proper in rat model of cyclophosphamide-induced neurotoxicity [99]. This histological improvement exhibited with SeNPs can be explained by their antioxidant, anti-inflammatory and anti-apoptotic activities depicted in our study. Hypervascularity and hypercellularity seen in the hippocampus and not in the prefrontal cortex can be interpreted by the ability of hippocampus to recover much better than the prefrontal cortex, since the CRS-induced hippocampal damage was less severe than that of prefrontal cortex.

In the present study, it is obvious that the effects of SeNPs were dose-dependent with rats treated with the 2.5 mg/kg dose exhibiting the best results in all studied parameters. Therefore, the dose–response curve for the effects of SeNPs in our study seems to be bell-shaped. Animal studies reported the occurrence of cytotoxicity with high doses of SeNPs and attributed such toxicity to their capability of inducing pro-oxidant effects and DNA damage [100]. This explains why the 5 mg/kg dose could not improve most of the studied parameters and even worsened some of them, such as the appearance of necrotic areas in the histological sections of the prefrontal cortex.

## Conclusion

To the best of our knowledge, the present study reported for the first time the neuroprotective role of SeNPs at a dose of 2.5 mg/kg in improving behavioral performance, prefrontal cortical and hippocampal serotonin level and histological architecture in rat model of CRS through their anti-oxidant, anti-inflammatory and anti-apoptotic effects. Finally, SeNPs appear to be a new promising pharmacological tool in protecting against the detrimental effects of chronic stress on the brain. The pharmacodynamics and toxicity of SeNPs on different organs need to be studied. The effect of SeNPs on brain neurogenesis and synaptogenesis in CRS need to be investigated as well.

## Data Availability

Data of this study are available from the corresponding author upon request.

## Abbreviations

BAX: Bcl-2-associated protein X
CRS: Chronic restraint stress
DLS: Dynamic light scattering
GPx: Glutathione peroxidase
GSH: Reduced glutathione
H&E: Hematoxylin and eosin
HP: Hippocampus
HPA: Hypothalamic pituitary adreno-cortical axis
KF-κβ: Nuclear factor kappa beta
MAPK: Mitogen activated protein kinase
MDA: Malondialdehyde
PDI: Polydispersity index
PFC: Prefrontal cortex
ROS: Reactive oxygen species
SeNPs: Selenium nanoparticles
TEM: Transmission electron microscope
TNF-α: Tumor necrosis factor alpha
